# Exploring the Zika virus epidemic’s association with fertility in a cohort of women of Northeastern Brazil: socioeconomic and educational gradients

**DOI:** 10.1590/1980-549720250044

**Published:** 2025-08-08

**Authors:** Carlos Sanhueza-Sanzana, Carl Kendall, Kasim Allel, Moisés Humberto Sandoval González, Rosa Livia Freitas de Almeida, Italo Wesley Oliveira Aguiar, Lívia Karla Sales Dias, Roberto Justa da Pires, Cristiane Cunha Frota, Francisco Gustavo Silveira Correia, Francisco Herlânio Costa Carvalho, Ivana Cristina de Holanda Cunha Barreto, Marto Leal, Anya Pimentel Gomes Fernandes Vieira-Meyer, George Rutherford, Ligia Kerr

**Affiliations:** I Universidade Federal do Ceará, Department of Community Health - Fortaleza (CE), Brazil.; II Tulane University, Celia Scott Weatherhead School of Public Health and Tropical Medicine, Department of Social, Behavioral, and Population Sciences - New Orleans, United States of America.; III University of Oxford, Nuffield Department of Primary Care Health Sciences - Oxford, United Kingdom.; IV Universidad de Chile, Doctor Fernando Monckeberg Barros Institute of Nutrition and Food Technology - Macul, Región Metropolitana, Chile.; V Universidade Federal do Ceará, Department of Pathology and Legal Medicine - Fortaleza (CE), Brazil.; VI Colégio Militar de Manaus - Manaus (AM), Brazil.; VII Fundação Oswaldo Cruz - Fortaleza (CE), Brazil.; VIII Universidade do Porto, Institute of Public Health - Porto, Portugal.; IX University of California, School of Medicine - San Francisco, California, United States of America.

**Keywords:** Zika virus infection, Fertility, Congenital zika syndrome, Health status disparities, Brazil, Infecção por zika vírus, Fertilidade, Síndrome congênita de zika, Desigualdades sociais, Brasil

## Abstract

**Objective:**

To explore the association between the Zika virus epidemic, fertility rates, and sociodemographic and behavioral factors influencing birth trends.

**Methods:**

A prospective cohort of 1,497 women aged between 15 and 39 years living in arbovirus-endemic areas in Fortaleza, Brazil, was analyzed. Women were enrolled in February 2018 and followed up two times every six months. The total fertility rate (TFR), age-specific fertility rate (ASFR), and mean age at first birth (MAB) were estimated and a multivariate Poisson regression model was used to explore the main factors associated with fertility.

**Results:**

The TFR was lowest during the epidemic period (2.64, 95%CI 2.06-3.06), increasing in the post-epidemic phase (TFR=3.52, 95%CI 3.18-3.86). Low educational attainment (RR=1.32, TFR=3.69, 95%CI 3.26-4.13), overcrowding (RR=1.27, TFR=3.26, 95%CI 2.98-3.54), and having undergone an abortion (RR=1.85, TFR=4.88, 95%CI 4.31-5.45) were associated with higher fertility rates. Conversely, having had an unwanted pregnancy was associated with reduced fertility (RR=0.81, TFR=2.65, 95%CI 2.41-2.89).

**Conclusion:**

We observed a slowdown in fertility rates during the epidemic period coincident with human Zika virus transmission with large differences by sociodemographic gradients.

## RESUMO

**Objetivo:**

Explorar a associação entre a epidemia do vírus zika (ZIKV), as taxas de fertilidade e os fatores sociodemográficos e comportamentais que influenciam as tendências de natalidade.

**Métodos:**

Analisamos uma coorte prospectiva de 1.497 mulheres com idade entre 15 e 39 anos de áreas endêmicas de arbovírus em Fortaleza, Brasil. As mulheres foram inscritas em fevereiro de 2018 e acompanhadas duas vezes depois, a cada seis meses. Estimamos a taxa de fertilidade total (TFT), a taxa de fertilidade específica por idade (ASFR) e a idade média ao primeiro parto (MAB), e usamos um modelo de regressão de Poisson multivariável para explorar os principais fatores associados à fertilidade.

**Resultados:**

A TFR foi menor durante o período epidêmico (2,64, intervalo de confiança de 95% - IC95% 2,06-3,06), aumentando na fase pós-epidêmica (TFR=3,52, IC95% 3,18-3,86). Baixo nível educacional (risco relativo - RR=1,32, TFR=3,69, IC95% 3,26-4,13), superlotação (RR=1,27, TFR=3,26, IC95% 2,98-3,54) e ter sofrido um aborto (RR=1,85, TFR=4,88, IC95% 4,31-5,45) foram associados a maiores taxas de fertilidade. Por outro lado, ter tido uma gravidez indesejada foi associado à fertilidade reduzida (RR=0,81, TFR=2,65, IC95% 2,41-2,89).

**Conclusão:**

Observamos desaceleração nas taxas de fertilidade durante o período epidêmico, coincidente com a transmissão humana do ZIKV com grandes diferenças por gradientes sociodemográficos.

## INTRODUCTION

In 2015, the Zika virus (ZIKV) outbreak in Brazil, initially detected in the state of Pernambuco, triggered a Public Health Emergency of National Importance (*Emergência em Saúde Pública de Importância Nacional* - ESPIN) declaration due to a surge in neonatal microcephaly cases^1^ ^,^ ^2^. The outbreak spread to 14 Brazilian states and 33 countries in the Americas^3^, resulting in 3,742 confirmed cases of congenital infections with abnormal child growth by March 5, 2024, with 1,828 (48.9%) attributed to Congenital Zika Syndrome (CZS), of which 1,380 (75.5%) occurred in the Northeast region^4^. ZIKV, alongside Dengue (DENV) and Chikungunya (CHIKV) viruses, circulated in urban areas, causing significant human morbidity and mortality^5^ ^,^ ^6^ ^,^ ^7^ ^,^ ^8^ ^,^ ^9^ ^,^ ^10^, with 11.6 million reported cases and 7,043 deaths between 2008 and 2019^11^.

While biological and epidemiological aspects of ZIKV and CZS are well-documented^5^ ^,^ ^6^ ^,^ ^7^ ^,^ ^8^ ^,^ ^9^ ^,^ ^10^, their demographic impacts remain less explored. Some studies suggest that fears of CZS and increased abortions led to delayed pregnancies, potentially contributing to declining birth rates^12^ ^,^ ^13^ ^,^ ^14^ ^,^ ^15^. For example, Brazil’s total fertility rate (TFR) dropped from 6.7 in 1960 to 1.7 in 2017^16^ ^,^ ^17^, though this decline varied regionally. The Southeast region reached replacement-level fertility between 1995 and 2000, while other regions maintained higher rates^16^. Sociodemographic factors, such as education, urbanization, healthcare access, and sterilization procedures were identified as drivers of this fertility transition^18^. In the state of Ceará, in northeastern Brazil, TFR decreased over time, reaching 1.70 per 100 thousand women of childbearing age in 2018, compared to the national average of 1.77^19^ ^,^ ^20^. In the present study, we investigate the association between the ZIKV epidemic, fertility rates, and sociodemographic and behavioral factors influencing birth trends in Ceará to elicit regional policymaking for future outbreaks.

## METHODS

### Study setting, design and population

A cohort of women of reproductive age, who lived in areas of high vulnerability for arboviruses infection in Fortaleza, state of Ceará, Brazil, were enrolled. The study sample was recruited from a prospective cohort study, *“ZIKA in Fortaleza (ZIF).”* Briefly, four primary health care units (PHCUs) within Fortaleza, most affected by arbovirus infections caused by CHIKV, were chosen to participate in ZIF. Two of these PHCUs are in the Barra do Ceará, an urban neighborhood located in the second-most densely populated area of Fortaleza^21^. This neighborhood has a very low Human Development Index (HDI=0.22), with poor infrastructure, lack of sanitation, difficulty accessing water and need for water storage, unpaved and poorly maintained streets and failure to collect household waste, all of which are favorable conditions for the proliferation of *Aedes aegypti* ^*22*^ ^,^ ^23^. The third PHCU is in the Esperança complex, in Fortaleza’s southwest area, with a population of 16,405 inhabitants (HDI=0.29). The fourth PHCU is in Rodolfo Teófilo, a more economically-advantaged town with 19,114 inhabitants (HDI=0.48)^23^ ^,^ ^24^. Women who attended any of the four PHCUs were enrolled in February 2018 and two follow-up waves were conducted, with an average follow-up time of six months ending in August 2019.

The study sampling criteria included women:

1. Living in the chosen PHCU area;
2. Aged between 15 and 39 years, due to a greater chance of becoming pregnant;
3. Being sexually active, defined as having at least one sexual relationship in the past 12 months;
4. Having no tubal ligation or health problem that might prevent pregnancy; and
5. Agreeing to participate in the study.

A probabilistic sample size of 1,497 women was estimated. More detailed information about sample calculation and design of the study is provided in a previous publication^10^.

### Main outcomes

To analyze fertility rates among the sampled women over time, the time variable was subdivided into three periods following the epidemiology of Zika in Brazil^24^ ^,^ ^25^:

1. Pre-epidemic period: from 2013 to 2014;
2. Epidemic period: from 2015 to 2016; and
3. Post-epidemic period: from 2017 to 2018.

“Fertility” for each woman was reconstructed using the birth history of each woman from 2013 on. In addition to the mother’s age and age at each birth, the authors thoroughly investigated up to five children reported born during the study recall period (2013-2018). Furthermore, we collected information about women’s pregnancies prior to 2013, the number of pregnancies that did not end in live births, and their pregnancy status at the time of interview. For all children, we collected information about sex, survival status, age (if alive), and age at death if deceased. *Age at first birth* was calculated as the difference between the birth date of the first-born child and the birth date of the women. *Mean age at first birth* (MAB) was defined as the average age at which the woman had her first pregnancy. To measure abortion, the questions “Have you had an abortion?” and “Have any of these abortions been induced?” were asked, using the WHO criteria^26^. By “spontaneous abortion,” the authors mean the involuntary termination of pregnancy, and “induced abortion” means the outcome of a deliberate interruption of pregnancy.

### Independent variables

Women responded to a closed-ended questionnaire with the following four major components. First, socioeconomic and demographic variables were the age of the participant, educational attainment, race/ethnicity, socioeconomic status according to Brazilian criteria (*Associação Brasileira de Estudos Populacionais* [Brazilian Association of Population Studies] - ABEP)^27^ ^,^ ^28^, employment status, whether women were receiving cash transfers from the *Bolsa Família* program (a cash transfer program of the Brazilian government) or any other state program, marital status, household food insecurity based on the instrument of the United States Department of Agriculture (USDA)^29^, the number of residents and children in the household, and poverty. Poverty was classified as income BRL≤179 per capita per month, or approximately USD54/month (using Mid-year 2017 BRL/USD exchange rate), USD1.80 per day. Second,household sanitation variables included sewage disposal, water source, and water storage. Third, sexual and reproductive health variables were use of condom in the last sexual intercourse, frequency of condom use, use of repellent to prevent ZIKV, knowledge of contraceptive methods, type of contraceptive method, age at first live birth, pregnancy history, desire for pregnancy, pregnancy postponement, abortion (spontaneous and/or induced), and the reason (if any) to postpone her pregnancy. Fourth, medical history variables were family history of arbovirus infections, self-reported comorbidities, diagnosed chronic conditions (e.g., hypertension, asthma, cardiovascular disease, and diabetes), and use of illicit drugs and tobacco.

### Statistical analysis

The authors reconstructed fertility trends of the sampled women, estimated differentials in fertility levels, and finally calculated the age-specific fertility rate (ASFR), TFR, and MAB for each age group, socioeconomic status (Brazilian ABEP criterion), self-reported race or skin color (white and non-white: Black, Indigenous, Asian, or mixed-race), and educational attainment (incomplete elementary school, complete elementary school, or higher education). Births in 2019 were censored due to the small number of births at the end of the study, which affected estimates, and because of the small proportion of women in the ZIF cohort who were waiting to give birth during the second wave of the study. For the study’s purposes, TFR, ASFR, and MAB rates were calculated for women between 15 and 39 years of age due to samples constrains. The equation and estimation approaches are described next.

First, the ASFR was calculated for five groups of five-year ranges: 15-19, 20-24, 25-29, 30-34, and 35-39 years of age (see Equation 1). ASFR represents the number of births occurring during a given year or a reference period per one thousand women-years of exposure for a specific age group. For each age group, where β_a_ denotes the number of births of women in age group *a* during the reference period^30^ ^,^ ^31^ ^,^ ^32^, and E_a_ denotes the number of women in age group *a* during the same reference period.

ASFRa=(βa/Ea)*1000 ( Equation 1 )

Second, the TFR was calculated, which is the number of children who would have been born per woman between 15 and 49 years of age if women were to pass through the childbearing years not subject to mortality (see Equation 2).

$$TFR=5*\sum_{a\epsilon A}ASFRa/1000\;\;$$ ( Equation 2 )

Third, MAB rates were computed, which consists in the average age at first birth at time (t), where b (_a_, t) denotes the age-specific birth rate for birth order one at (single) age *a* and time t, and *a* max denotes the highest age at which first births are observed (see Equation 3).

$$MAB\left(t\right)=\frac{\sum_{0}^{amax}\left(a+0.5\right)b(a,t)}{\sum_{0}^{amax}b(a,t)}$$ ( Equation 3 )

For the present study, TFR, ASFR, and MAB rates were calculated for women between 15 and 39 years of age due to the small number of women ≥40 years in the sample.

Finally, univariable (crude) and multivariate (adjusted) analyses were employed using Poisson regressions. The outcome variable was the number of children born by women of reproductive age. Risk ratios (RR) and their respective 95% confidence intervals (95%CI) were reported. The variables that were significant at p-value<0.05 univariately were adjusted in a final multivariate regression model (Table 1). All models were tested for multicollinearity using the variance inflation factor (VIF) and variables were kept if VIF<5.

**Table 1.** Age Specific Fertility Rate of women between 15 and 39 years of age from the “Zika in Fortaleza” cohort and between 2013 and 2018.

Variable (n=1,497)	2013-2014		2015-2016		2017-2018		p-value
	ASFR	95%CI	ASFR	95%CI	ASFR	95%CI	
Educational attainment							
Incomplete elementary school by age group							
15-19	0.21	0.15-0.28	0.26	0.19-0.33	0.18	0.12-0.23	<0.001
20-24	0.25	0.16-0.33	0.17	0.10-0.24	0.22	0.15-0.29	
25-29	0.13	0.07-0.20	0.09	0.04-0.15	0.18	0.11-0.26	
30-34	0.08	0.02-0.13	0.07	0.02-0.12	0.10	0.04-0.16	
35-39	0.15	0.01-0.36	0.03	0.01-0.09	0.08	0.03-0.14	
TFR	4.21	2.93-5.48	3.24	2.57-3.92	3.89	3.17-4.61	
Complete elementary school or higher education by age group							
15-19	0.09	0.06-0.11	0.09	0.07-0.11	0.14	0.11-0.18	<0.001
20-24	0.12	0.09-0.15	0.13	0.10-0.16	0.16	0.13-0.20	
25-29	0.11	0.08-0.14	0.10	0.07-0.13	0.17	0.13-0.21	
30-34	0.06	0.03-0.09	0.10	0.06-0.13	0.13	0.09-0.17	
35-39	0.08	0.00-0.13	0.06	0.02-0.10	0.05	0.02-0.08	
TFR	1.93	1.64-2.22	2.45	2.08-2.83	3.38	3.00-3.77	
Socioeconomic Status (SES)							
Low SES (D, E) by age group							
15-19	0.20	0.12-0.28	0.28	0.18-0.38	0.22	0.12-0.33	<0.001
20-24	0.15	0.07-0.22	0.18	0.09-0.26	0.24	0.15-0.33	
25-29	0.14	0.07-0.22	0.14	0.08-0.21	0.17	0.10-0.25	
30-34	0.01	0.01-0.04	0.10	0.02-0.17	0.09	0.03-0.16	
35-39	0.18	0.01-0.54	0.03	0.01-0.09	0.06	0.01-0.13	
TFR	3.52	1.58-5.45	3.75	2.86-4.64	4.07	3.15-5.00	
High SES (A, B1, B2, C) by age group							
15-19	0.10	0.08-0.12	0.11	0.08-0.13	0.14	0.11-0.18	<0.001
20-24	0.14	0.11-0.17	0.13	0.10-0.16	0.16	0.13-0.19	
25-29	0.11	0.08-0.14	0.09	0.06-0.11	0.17	0.13-0.21	
30-34	0.07	0.04-0.10	0.09	0.06-0.12	0.13	0.09-0.16	
35-39	0.03	0.00-0.10	0.06	0.01-0.10	0.06	0.03-0.09	
TFR	2.38	1.92-2.84	2.45	2.10-2.80	3.44	3.07-3.81	

ASFR: Age Specific Fertility Rate; TFR: Total Fertility Rates; p-value significance statistics=<5%, Poisson Regression Model; 95%CI: 95% confidence interval.

All data analyses were processed using the statistical package Stata v.17 (Stata Corporation, College Station, Texas, USA). Specifically, the fertility estimation method proposed by Schoumaker (2013) and the “*tfr2*” module for fertility rates were used.

### Data Availability Statement

The data and codes used for the analysis of this study are available for public access with prior authorization from the principal investigator, Dr. Ligia Kerr, at the following link: https://github.com/kasimallel/Zikavirus_Fertility_Brazil/blob/main/README.md

## RESULTS

### Characterization of women’s sociodemographic profile

Of the 1,497 eligible women from the ZIF study, 1,175 women (78.4% of the first wave) completed the second wave of the study. In Table 2, we show the descriptive statistics of our final sample (1,497 women). Most women were under 29 years of age and had complete elementary education or higher (74.4%). Most women were non-white (88.6%), belonged to the middle or higher socioeconomic class (84.6%, A/B/C), 77.3% reported not working or being unemployed, 19.2% were extremely poor, and 29.1% lived on less than one minimum wage per month (equivalent to USD 170, or USD 5.70/day). Most participants received federal cash transfers, did not store water, and had one or two children (58%) by the age of 18 (35.2%) or 19-24 years (30.8%). One-third (31.8%) reported using condoms at the last sexual intercourse.

**Table 2.** Descriptive characteristics of women between 15 and 39 years of age from the “Zika in Fortaleza” cohort, Fortaleza (CE), Brazil, 2018.

Variable n=1,497	n	%
Age group, years (n=1,497)		
15-19	310	20.7
20-24	418	27.9
25-29	325	21.7
30-34	256	17.1
35-39	188	12.5
Educational attainment (n=1,496)		
Incomplete elementary school	383	25.6
Complete elementary school or higher education	1,113	74.4
Race or color of skin (n=1,487)		
White	170	11.4
Non-white	1,317	88.6
Socioeconomic status, ABEP criterion (n=1,497)		
Higher (A, B and C)	1,267	84.6
Lower (D and E)	230	15.4
Employment situation (n=1,497)		
Formal job	133	8.9
Informal job	206	13.8
Does not work or is unemployed	1,158	77.3
Beneficiary of the *Bolsa Família* program (n=1,497)		
Not a beneficiary	674	45.0
Beneficiary	823	55.0
Number of children (n=1,497)		
No children	428	28.6
One to two	869	58.0
Three or more	200	13.4
Used a condom in the last sexual intercourse (n=1,489)		
Yes	474	31.8
No	1,015	68.2
Use of condom frequency (n=1,488)		
Always	318	21.4
Sometimes	618	41.5
Never	552	37.1
Wanted to get pregnant (n=1,475)		
Yes	562	38.1
No	913	61.9
Had abortion (n=1,493)		
No	900	60.3
Yes	254	17.0
No history of pregnancy	339	22.7

At the time of the second interview, 77.3% of the participants reported they were pregnant, and 17% had a history of induced or spontaneous abortion. Only 2.1% reported postponing pregnancy for fear of ZIKV infection, while 14.1% reported postponing pregnancy due to an unfavorable economic situation. More than half (59.6%) had family histories of DENV, CHIKV, or ZIKV infections. Finally, a minority of women had comorbidities (20.6%) and had not used drugs (13.9%) or smoked tobacco (32.7%).

### Fertility rates and mean age of first birth

**Figure 1.**
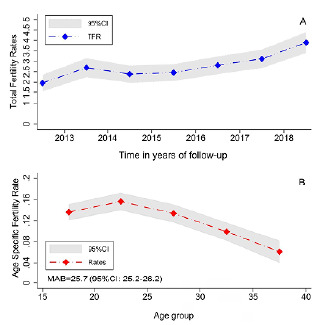
Total fertility rates* and age specific fertility rates^†^ of women aged 15 to 39 years from the “Zika in Fortaleza (CE)” cohort, reported between 2013 and 2018^‡^.

We estimated a TFR of 2.9 births per 100 thousand women-years (95%CI 2.7-3.1) for the six years preceding the initiation of the cohort study. We observed a sustained increase between 2016 and 2018; from 2.8 (95%CI 2.4-3.2) to 3.8 (95%CI 3.4-4.4) (p<.001). The TFR during the epidemic ZIKV period experienced a slowdown relative to the pre-epidemic period and subsequently increased during the post-epidemic period (Table 3; Figure 1A). The TFR for 2013 was already 2.7; in 2014, we estimated a TFR of 2.4, and 2.5 in 2015; however, the TFR increased again in the post-epidemic period. In this sense, there was a slowdown in fertility rates compared to the pre-epidemic period, but there was stabilization during the epidemic years, followed by a subsequent increase in the TFR in the period following the peak of the ZIKV epidemic.

**Table 3.** Total Fertility Rates from the “Zika in Fortaleza” cohort according to their socioeconomic status and educational attainment.

Variable (n=1,497)	2013-2014		2015-2016		2017-2018		p-value
	TFR	95%CI	TFR	95%CI	TFR	95%CI	
Educational attainment							
Complete elementary school or higher	1.9	1.6-2.2	2.5	2.1-2.8	3.4	3.0-3.8	<0.001
Incomplete elementary school	4.2	2.9-5.5	3.2	2.6-4	3.9	3.2-4.6	
Socioeconomic status							
High (A-B and C)	2.4	1.9-2.8	2.4	2.1-2.8	3.4	3.1-3.8	<0.001
Low (D and E)	3.5	1.6-5.5	3.8	2.9-4.6	4.1	3.2-5	
Race or skin color							
White	1.5	0.8-2.1	2.4	1.5-3.3	3.9	2.9-5	0.310
Non-white	2.8	2.1-3.4	2.7	2.3-3	3.5	3.1-3.8	
*Bolsa Família* program							
Not a beneficiary	1.4	1.1-1.7	1.9	1.4-2.3	3.5	2.9-4	<0.001
Beneficiary	3.5	2.6-4.3	3.3	2.8-3.8	3.6	3.1-4	
Household food insecurity							
No	1.9	1.5-2.2	2.3	1.8-2.8	3.6	3.0-4.1	<0.001
Yes	3.2	2.3-4.0	3.2	2.6-3.7	4.2	3.6-4.8	
Loss to follow-up	2.2	1.6-2.8	2.2	1.6-2.7	2.3	1.7-2.9	
Household residents							
Three or less	1.6	1.3-2	2.4	1.9-2.8	3.3	2.8-3.8	<0.001
Four or more	3.4	2.5-4.2	2.9	2.4-3.3	3.7	3.2-4.1	
Poverty situation							
Yes	3.4	2.3-4.5	4.2	3.2-5.2	4.9	3.9-5.8	<0.001
No	2.3	1.7-2.9	2.2	1.9-2.6	3.6	3.1-3.9	
Water Storage							
Yes	2.7	2.1-3.3	3.1	2.5-3.8	3.5	3.1-3.9	<0.001
No	2.5	1.8-3.2	2.5	2.1-2.8	3.60	2.9-4.3	
Used a condom in the last sexual intercourse							
Yes	3.5	1.8-5.3	2.6	1.9-3.3	3.10	2.5-3.7	<0.001
No	2.2	1.9-2.5	2.7	2.3-3.0	3.66	3.2-4.1	
Wanted to get pregnant							
No	3.3	2.3-4.3	2.2	1.8-2.5	2.79	2.4-3.2	<0.001
Yes	1.7	1.3-2.0	3.3	2.7-3.9	4.80	4.2-5.5	
TFR	2.6	2.1-3.1	2.6	2.3-3	3.52	3.2-3.9	<0.001

TFR: Total Fertility Rates; 95%CI: 95% confidence intervals.

*Rates computed per each year. Constant age fertility schedule was assumed. ^†^Age Specific Fertility Rates computed by 5-year periods; ^‡^MAB: Mean age at first birth. TFR: Total Fertility Rates; 95%CI: 95% confidence interval.

The ASFR was higher in the groups between 20 and 24 years of age (0.15 births per 100 thousand women-years), followed by the group of 15-19 years (ASFR=0.13). The ASFR declined in older women, particularly in the 30-34 (ASFR=0.09, 95%CI 0.08-0.11) and 35-39 age group (ASFR=0.06, 95%CI 0.03-0.08) (Figure 1B).

**Figure 2.**
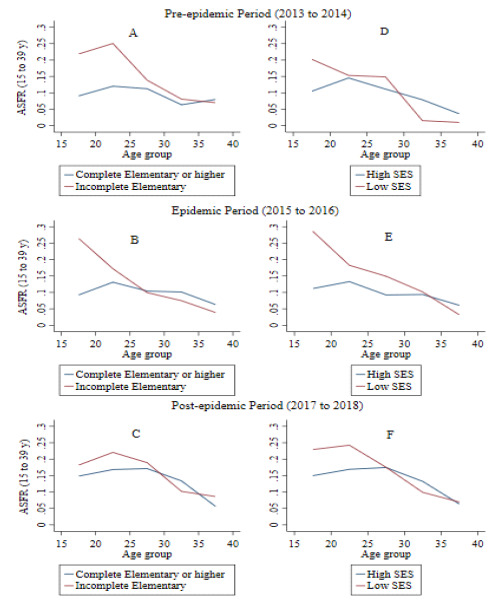
Age Specific Fertility Rates* by socioeconomic status and educational attainment for women aged 15 to 39 years from the “Zika in Fortaleza (CE)” cohort between 2013 and 2018.

Differences in fertility rates were observed by women’s socioeconomic status in all periods studied, but particularly during the epidemic period. Women in socioeconomic classes D and E had higher fertility (TFR=3.8, 95%CI 2.9-4.6) compared to women of higher socioeconomic status (TFR=2.5 births per one thousand women, 95%CI 2.1-2.8) (Table 3; Figure 2). A similar situation occurred when comparing the MAB, suggesting that women from high socioeconomic status became pregnant at a later age (26 years, 95%CI 25.4-26.6), compared to women from lower socioeconomic status (MAB=24.2, 95%CI 23.1-25.4) (Figure 2).

*Rates computed per each year. ASFR: Age Specific Fertility Rate, computed by each year. Constant age fertility schedule was assumed; SES: Socioeconomic status.

There was a significant difference in fertility between women who were beneficiaries of the *Bolsa Família* program and women who experienced household food insecurity. We observed that female beneficiaries of the *Bolsa Família* program during the epidemic period had a TFR of 3.3 (95%CI 2.8-3.8) compared to non-beneficiaries (TFR=1.9, 95%CI 1.4-2.3; p<0.001). In turn, women who experienced food insecurity had a TFR of 3.2 (95%CI 2.6-3.7) compared to women who did not (p<0.001, Table 3). Overall, according to our findings, poor women had a TFR of 4.2 (95%CI 3.2-5.2) compared to less poor women (TFR=2.2, 95%CI 1.9-2.6). Women who had their first child before the age of 18 had higher fertility rates in the epidemic period (TFR=3.5, 95%CI 2.9-4.2) compared to women who had their first child after (2.83, 95%CI 2.3-3.3, Table 3).

Women having higher educational attainment had a lower fertility rate during the epidemic period (TFR=2.5, 95%CI 2.1-2.8) per one thousand women, compared to women with less educational attainment (TFR=3.2, 95%CI 2.6-3.9) (Table 3). Women with less educational attainment have had their children earlier, but also stopped having children earlier.

### Social gradients and factors associated with fertility

According to our Poisson univariate regression analyses, fertility rates increased with lower educational attainment or socioeconomic status, unemployment, receiving cash transfers, higher number of people living in the household, and women’s age at the first live birth or whether they experienced abortion.

In our adjusted results we found that age and educational attainment are strong predictors of fertility (Table 4). Younger women between 15 and 19 years of age had 3.78 times higher adjusted odds of fertility, and women between 20 and 24 years had 3.23 times higher adjusted odds of fertility compared to older women (35-39 years, p<0.01) (Table 4). Those women from lower educational attainment status had 1.32 times higher adjusted odds of fertility compared to women with higher educational attainment (p<0.01). Women who lived with four or more people at the household had 1.22 times higher adjusted odds of fertility than those who lived with less than four people (p<0.01). The desire for pregnancy was associated with 19% lower adjusted odds of fertility (p<0.01).

**Table 4.** Poisson regression multivariate analysis of Total Fertility Rates in women from the “Zika in Fortaleza” cohort between 2013 and 2018.

Variable (n=1,497)	Model 1			Model 2*		
	RR (crude)	95%CI	p-value	RR (adjusted)	95%CI	p-value
Age group						
35-39	1		<0.001	1		<0.001
30-34	1.7	1.2-2.5		1.9	1.3-2.7	
25-29	2.6	1.7-4		2.6	1.7-4.0	
20-24	3.2	1.9-5.2		3.2	1.9-5.5	
15-19	3.7	2.2-6.4		3.7	2.2-6.3	
Educational attainment						
Complete elementary school or higher education	1	1	<0.05	1	1	<0.001
Incomplete elementary school	1.2	1.2-2.5		1.32	1.1-1.5	
Socioeconomic status, ABEP criterion						
Higher (A, B and C)	1	1	<0.05			
Lower (D and E)	1.2	1-1.4				
Household residents						
Three or less	1	1	<0.001	1	1	<0.001
Four or more	1.3	1.1-1.4		1.23	1.1-1.4	
Contraceptive methods						
Condom or birth control pills	1	1	<0.001			
Long-term	1.4	1.2-1.6				
Do not use	1.2	1.0-1.4				
Wanted to get pregnant						
Yes	1	1	<0.001	1	1	<0.001
No	0.8	0.74-0.9		0.8	0.7-0.9	
Had an abortion						
No	1		<0.001	1	1	<0.001
Yes	1.8	1.6-2.1		1.9	1.6-2.1	

*Model adjusted for the reproductive and sexual health variable and socioeconomic and demographic variables; 95%CI: 95% confidence interval; RR: risk ratios.

## DISCUSSION

According to our results, the decline in fertility rates during the Zika epidemic in Brazil is evident. We demonstrated a strong association between the epidemic ZIKV period and fertility, in line with previous literature^12^ ^,^ ^13^ ^,^ ^14^ ^,^ ^15^. We observed a slowdown in fertility rates among women in the ZIF-cohort between 2015 and 2016, followed by an increase post-epidemic. Higher fertility rates in the last period may be due to age censoring. These findings support our hypotheses: women postponed pregnancy due to ZIKV risks, and fertility differs by socioeconomic status and level of education.

The observed slowdown of the fertility rate is consistent with recent studies carried out in the Northeast region, according to which there were 36,546 fewer births than expected^12^ ^,^ ^13^ ^,^ ^15^. This deficit in births showed important differences between highly and less affected regions by ZIKV, which demonstrates a potential association between the deficit of births and ZIKV and CZS cases. This decline in the TFR is also consistent with the official data on the number of live births in Fortaleza, where 39,548 live births occurred in 2015, dropping to 37,463 in 2016^33^. The estimated TFR for Fortaleza was 44.5 in 2015, decreasing to 41.2 in 2016, indicating a decline in fertility during the epidemic period, as we show in the present study^34^.

TFRs were higher among poorer and less educated women; they were also higher at younger ages and declined as women approached 30 years of age. For poorer and less educated women in our cohort, pregnancy occurs earlier and more often than for richer and more educated women. Conversely, richer and more educated women postponed their pregnancy until the age of 30 years.

The poorest and less educated women and their infants were the groups most affected by the ZIKV epidemic and CZS. This phenomenon was produced by a range of factors including geography, class, and race, as demonstrated by the larger number of cases of microcephaly and infant deaths reported in the Northeastern states of Brazil. This is a product of inequity in the life conditions of these women, mainly Black and mixed-race women from low-income families^10^ ^,^ ^35^ ^,^ ^36^ ^,^ ^37^.

Researchers have reported the effects of the ZIKV epidemic on shaping the reproductive intentions of women in Brazil^13^ ^,^ ^14^ ^,^ ^38^. In addition, they also show an increasing racial vulnerability: Black and mixed-race women were more likely to postpone pregnancy than white women. We found very different results: 61.9% did not want to get pregnant, regardless of the ZIKV epidemic, and only 2.1% reported avoiding pregnancy due to the fear of ZIKV infection and CZS. Moreover, 17.1% of the women reported abortion, and 14% of these were reported as induced abortions.

We identified significant fertility disparities between women with low levels of education and socioeconomic status versus those with high levels of education and wealth. These disparities, influenced by race, gender, and class, highlight the need for sexual and reproductive health policies focused on human rights for women^39^ ^,^ ^40^ ^,^ ^41^. These findings are consistent with studies showing higher ASFR in younger women with lower levels of wage income^14^ ^,^ ^15^.

Many “emerging” countries in a post-transitional fertility regime experienced a decline in TFR below 1.72 children per woman^42^ ^,^ ^43^. This group includes all countries and regions of the world, except for Sub-Saharan Africa. Almost all countries in Latin America, with few exceptions (Bolivia, Guatemala, and Haiti), demonstrate decline along with improvements in women’s education and participation in the labor market^42^. However, according to the literature, fertility changes occur in response to shocks and as a consequence of economic crisis^12^ ^,^ ^42^ and public health emergencies^44^.

We highlight issues regarding contraceptive access, reflecting women’s autonomy and rights. Women using long-term methods, such as IUDs or diaphragms, had higher fertility than those using pills or condoms. Often older, they likely sought for more reliable birth control after completing their reproductive careers^14^ ^,^ ^15^.

While contraceptive methods are associated with fertility, we know that the economy plays a large role in the intendedness of pregnancy. We found that almost one third of the women in the ZIF-cohort were formally unemployed and more than two fifth of the sample did not work. This shows the diminished financial autonomy of these women as they were out of the labor market, degrading their ability to generate income associated with fertility and poverty’s intergenerational transmission^14^ ^,^ ^15^ ^,^ ^45^ ^,^ ^46^.

Brazilian women face vulnerabilities due to high maternal mortality, adolescent pregnancies, and limited access to legal abortion. These outcomes highlight inequality, poverty, and discrimination against Indigenous, Black, and adolescent women^17^ ^,^ ^47^. The intersection of gender, race, and education increases pregnancy risks. In many Latin American countries, including Brazil, El Salvador, and Honduras, abortion remains illegal, forcing secrecy and causing severe consequences, including death^44^ ^,^ ^48^ ^-^ ^52^.

Another expression of unequal power relationships is that adolescent pregnancy is high in vulnerable groups. Lower wealth quintiles, with less education, have more desired children and fewer unwanted pregnancies, while higher quintiles experience the opposite, leading to more unwanted pregnancies^39^ ^,^ ^40^ ^,^ ^41^. In contrast, authors of other studies have explained that poor pregnant adolescents in Latin America report higher levels of unintended pregnancy^17^ ^,^ ^47^. As expected, the answer to our research questions forces a discussion of a wide range of individual, collective, cultural, and structural factors that influence fertility, beyond contraception.

In this study, we showed that the ZIKV epidemic impacts fertility, with trends varying according to education and socioeconomic class. These factors highlight social inequalities in women’s sexual and reproductive health in arbovirus-endemic areas of northeastern Brazil.

### Limitations

The present study has some limitations. While most studies on fertility use the age range of 15 to 49, our cohort included women 15-39, affecting absolute fertility values. We focused on childbearing-age women, especially those at higher risk of congenital Zika syndrome. Given the fertility decline after the age of 39, we prioritized higher fertility rates and excluded women aged 40 years or older.

Additionally, over 40% of the participating women had ZIKV infection, which further increased the risk of suffering the consequences of this infection for both women and their children. Still, the trend data are strongly indicative of the effect of ZIKV on fertility. Finally, a number of important variables concerning food insecurity that might affect fertility were only collected in the second wave, and retrospectively with regard to the time of the first wave, which could introduce misclassification bias.
